# Response to everolimus is seen in TSC-associated SEGAs and angiomyolipomas independent of mutation type and site in *TSC1* and *TSC2*

**DOI:** 10.1038/ejhg.2015.47

**Published:** 2015-03-18

**Authors:** David J Kwiatkowski, Michael R Palmer, Sergiusz Jozwiak, John Bissler, David Franz, Scott Segal, David Chen, Julian R Sampson

**Affiliations:** 1 Brigham and Women's Hospital, Boston, MA, USA; 2 Novartis Oncology Translational Medicine, Cambridge, MA, USA; 3 The Children's Memorial Health Institute, Warsaw, Poland; 4 St Jude Children's Research Hospital, University of Tennessee Health Science Center, Memphis, TN, USA; 5 Cincinnati Children's Hospital Medical Center, Cincinnati, OH, USA; 6 Novartis Pharmaceuticals Corporation, East Hanover, NJ, USA; 7 Institute of Medical Genetics, School of Medicine, Cardiff University, Cardiff, UK

## Abstract

Tuberous sclerosis complex is an autosomal dominant disorder that occurs owing to inactivating mutations in either *TSC1* or *TSC2*. Tuberous sclerosis complex-related tumors in the brain, such as subependymal giant cell astrocytoma, and in the kidney, such as angiomyolipoma, can cause significant morbidity and mortality. Recently, randomized clinical trials (EXIST-1 and EXIST-2) of everolimus for each of these tuberous sclerosis complex-associated tumors demonstrated the benefit of this drug, which blocks activated mammalian target of rapamycin complex 1. Here we report on the spectrum of mutations seen in patients treated during these trials and the association between mutation and response. *TSC2* mutations were predominant among patients in both trials and were present in nearly all subjects with angiomyolipoma in whom a mutation was identified (97%), whereas *TSC1* mutations were rare in those subjects (3%). The spectrum of mutations seen in each gene was similar to those previously reported. In both trials, there was no apparent association between mutation type or location within each gene and response to everolimus. Everolimus responses were also seen at a similar frequency for the 16–18% of patients in each trial in whom no mutation in either gene was identified. These observations confirm the strong association between *TSC2* mutation and angiomyolipoma burden seen in previous studies, and they indicate that everolimus response occurs regardless of mutation type or location or when no mutation in *TSC1* or *TSC2* has been identified.

## Introduction

Tuberous sclerosis complex (TSC) is an autosomal dominant disorder that occurs as a result of inactivating mutations in either *TSC1* or *TSC2*, and affects about 1 in 10 000 individuals worldwide.^1^ It is characterized by prominent neurodevelopmental features and by tumors that develop in the brain, skin, heart, kidneys and lungs.^1^

The brain tumor seen most commonly in TSC is the subependymal giant cell astrocytoma (SEGA), which is usually located near the foramen of Monro and develops in as many as 20% of individuals with TSC.^2^ When they grow to be of significant size, SEGAs cause significant morbidity through ventricular obstruction, leading to hydrocephalus and compression of nearby brain structures.^2, 3^ In the past, surgical resection had been the main treatment approach for these tumors, but both postoperative morbidity and recurrence after surgery are important clinical issues; this has led to the use of rapamycin and/or everolimus for the treatment of SEGAs.^2, 4, 5, 6, 7, 8, 9^

Renal angiomyolipomas are seen in about 80% of individuals with TSC; they cause the largest share of adult deaths from TSC, through increased risk of hemorrhage and loss of functional renal parenchyma leading to chronic kidney failure.^10, 11, 12^ Both surgical resection and therapeutic embolism have been used to control these tumors, and nephron-sparing approaches are considered mandatory.

More than 2000 nonsynonymous mutations have been identified in the *TSC1* and *TSC2* genes (http://chromium.liacs.nl/LOVD2/TSC/home.php).^13, 14, 15, 16, 17, 18, 19, 20, 21, 22, 23, 24, 25, 26, 27, 28, 29, 30, 31, 32^ In both *TSC1* and *TSC2*, 50–60% of all mutations are single base substitution mutations, and a large fraction of those are C to T transitions at CpG sites – likely due to deamination of a methylated C residue. In *TSC1* the majority of the C to T transitions cause nonsense mutations, whereas in *TSC2* both nonsense and missense mutations occur owing to this mechanism. Insertion and deletion mutations of size 1–4 nucleotides are also quite common in both *TSC1* and *TSC2*, and usually occur at sites of short repetitive sequences. Large genomic deletions and rearrangements in the *TSC2* gene are also relatively common and are seen in about 6% of unselected TSC patients.^30^ In contrast, large genomic deletions are quite rare in *TSC1* and are seen in only about 0.5% of unselected cases.^1, 30^ This may be due, in part, to a sequence within the adjacent *PKD1* gene that blocks the replication fork and could lead to double-strand breaks in this region of chromosome 16^34^. Ten to fifteen percent of TSC patients have no mutation identified in these genes, despite a careful search, and it seems likely that mosaicism accounts for a significant fraction of those without an identified mutation.

A summation of published reports on mutation identification in TSC^13, 14, 15, 16, 17, 18, 19, 20, 21, 22, 23, 24, 25, 26, 27, 28, 29, 30, 31, 32^ indicates that among patients with identified mutations, about 21% occur in *TSC1* and 79% occur in *TSC2*.^33^ This difference in mutation frequency in the two genes is likely due, in part, to the larger size of the coding region of *TSC2* (5.4 kb) compared with *TSC1* (3.5 kb), and it also appears to be due to an intrinsic difference in mutation rate given that about two-thirds of all cases of TSC are a result of new mutations.

EXIST-1 and EXIST-2 were multicenter, randomized, placebo-controlled clinical trials examining the benefit of everolimus for the treatment of SEGAs and angiomyolipoma, respectively.^34, 35^ Subjects with SEGAs in EXIST-1 also had to have a diagnosis of TSC, whereas those with angiomyolipoma in EXIST-2 could have either TSC or sporadic lymphangioleiomyomatosis (LAM). In this article, we report on an analysis of *TSC1* and *TSC2* mutations in the subjects participating in the two trials.

## Materials and methods

### Patient recruitment and clinical results of the EXIST trials

Detailed reports on patient recruitment and conduct of the EXIST trials have been published elsewhere.^34, 35^

### Mutation analysis

Venous blood was collected in EDTA tubes from all patients (who had given informed consent) and sent to Quest Diagnostics (San Juan Capistrano, CA, USA) for genomic DNA extraction using the Gentra Systems Autopure LS (Minneapolis, MN, USA). Full exonic sequencing for *TSC1* and *TSC2* was performed on samples from the EXIST-1 trial at Novartis Pharmaceuticals Corporation Inc. (Cambridge, MA, USA). Amplification was performed using Advantage HF 2 PCR Kit (Clontech Laboratories Inc., Mountain View, CA, USA) with M13-labeled primers. Primer sequences are available upon request from MRP (michaelr.palmer@novartis.com).

PCR purification and standard M13-primered bidirectional Sanger sequencing was performed at GeneWiz Inc. (Cambridge, MA, USA). Sequence analysis was performed at Novartis Pharmaceuticals Corporation Inc., using Mutation Surveyor (SoftGenetics LLC, State College, PA, USA), with independent read confirmation. *TSC1* and *TSC2* duplication and/or deletion analysis was performed at Novartis Pharmaceuticals Corporation Inc., using MRC-Holland's P124 TSC1 and P046-B2 TSC2 MLPA Kits (MRC-Holland, Amsterdam, The Netherlands), according to the manufacturer's instructions with 100 ng of DNA input. MLPA analysis was performed using GeneMarker Software (SoftGenetics LLC) with its MLPA analysis package. *TSC2* exon 7 was excluded from analysis owing to high levels of variability in its amplification peak relative to other probe sets. A DNA sample was required to have at least two consecutive probe sets in the duplication and/or deletion range to be considered positive. All samples were run two times for confirmation. Sequencing and duplication/deletion analysis for the EXIST-2 trial was performed using very similar PCR conditions and assays at Athena Diagnostics Inc. (Worcester, MA, USA), using its Complete Tuberous Sclerosis Evaluation panel. This analysis consists of Sanger sequencing of all exons of *TSC1* and *TSC2*, and the same MLPA duplication and deletion assays for *TSC1* and *TSC2* described above.

### Mutation definition and assessment of pathogenicity

The term mutation is used here to mean a sequence variant that is known or thought to ablate the function of the *TSC1* or *TSC2* gene transcript or protein product. We used several criteria to assess whether sequence variants were mutations. First, chain-terminating (nonsense and out-of-frame indels) and splice variants affecting consensus nucleotides were considered mutations. Second, missense and in-frame deletion variants were compared with those reported in the Leiden Open Variation Database (LOVD) for Tuberous Sclerosis Complex (http://chromium.liacs.nl/LOVD2/TSC/home.php), and information indicative of likely pathogenic significance was used to confer mutation status, when available. Third, in the remaining missense variant cases, the amino-acid Block Substitution Matrix^36^ was used to assess the importance of missense changes on function. Missense variants with a score ≤−1 were considered mutations.

### Nomenclature and database information

Exon numbering for *TSC1* and *TSC2* was according to that used by the LOVD (http://chromium.liacs.nl/LOVD2/TSC). All variant data were submitted to that database.

### Statistical analysis

Using GraphPad software (http://graphpad.com/quickcalcs), categorical variables were compared using the *χ*^2^ test for comparisons with an expected distribution and Fisher's exact test for comparisons between two sets of observations. *P*-values reported are nominal. No multiplicity adjustments were made, so statistical interpretation should be made with caution.

## Results

### Mutation findings in the EXIST-1 and EXIST-2 trials

In the EXIST-1 trial, 117 patients with SEGA and TSC were randomly assigned to everolimus (*n*=78) or placebo (*n*=39).^34^ The median age of subjects was 9.5 years (range 0.8–26.6 years). Twenty-seven (35%) of 78 patients in the everolimus group and zero of 39 in the placebo group had a response in terms of a reduction in the total SEGA volume of ≥50%.^34^

DNA samples from 116 patients were available for mutation analysis; 97 of 116 (84%) samples were found to have a mutation in either *TSC1* or *TSC2* (Table 1 and Supplementary Table 1). The distribution of mutation types was similar to that reported previously.^33^ However, only 13 (13%) of patients with defined mutations had mutations in *TSC1*, whereas 84 (87%) had mutations in *TSC2*. This is a somewhat lower proportion of *TSC1* mutations than was seen in other series, in which 21% had *TSC1* mutations and 79% had *TSC2* mutations (*P*=0.0662, *χ*^2^ test).^33^ Nineteen of 116 (16%) had no mutation identified; this was similar to multiple previous studies.^13, 14, 15, 16, 17, 18, 19, 20, 21, 22, 23, 24, 25, 26, 27, 28, 29, 30, 31, 32^

In the EXIST-2 trial, 118 patients with angiomyolipoma, and TSC and/or LAM, were randomly assigned to receive everolimus (*n*=79) or placebo (*n*=39).^35^ The median age of subjects was 31 years (range 18.0–61.0 years); 78% (92 of 118) of patients had angiomyolipomas in both kidneys, 29% (34 of 118) had an angiomyolipoma of at least 8 cm in its longest dimension and nearly 40% (46 of 118) had a previous intervention, including 19% (22 of 118) with prior nephrectomy. Of patients receiving everolimus, 42% (33 of 79) showed a response at 12 weeks *vs* 0% (0 of 39) of those receiving placebo.^35^ Response was defined as a reduction in angiomyolipoma volume (sum of volumes of all target angiomyolipomas identified at baseline) of 50% or more relative to baseline and absence of angiomyolipoma progression.

Mutation analysis was completed on the DNA samples of 114 patients participating in EXIST-2 (109 from those with TSC and 5 from those with LAM and angiomyolipomas but not TSC). No mutations were identified in the non-TSC subjects. The proportion of subjects with TSC who had mutations identified and the distribution of mutation types was similar in both the EXIST-2 and EXIST-1 populations (Table 2 and Supplementary Table 2). However, the proportion of patients with *TSC1* mutations in EXIST-2 (three patients, 3%) was much smaller than that in EXIST-1 (13 patients, 13%) (*P*=0.0178, Fisher's exact test two-tailed). Furthermore, the observed distribution of *TSC1* vs *TSC2* mutations in EXIST-2 was significantly different than the aggregate mutation data (*P*<0.0001, *χ*^2^ test).^33^

### Correlation between mutation site and type and response to everolimus

**Figure 1:**
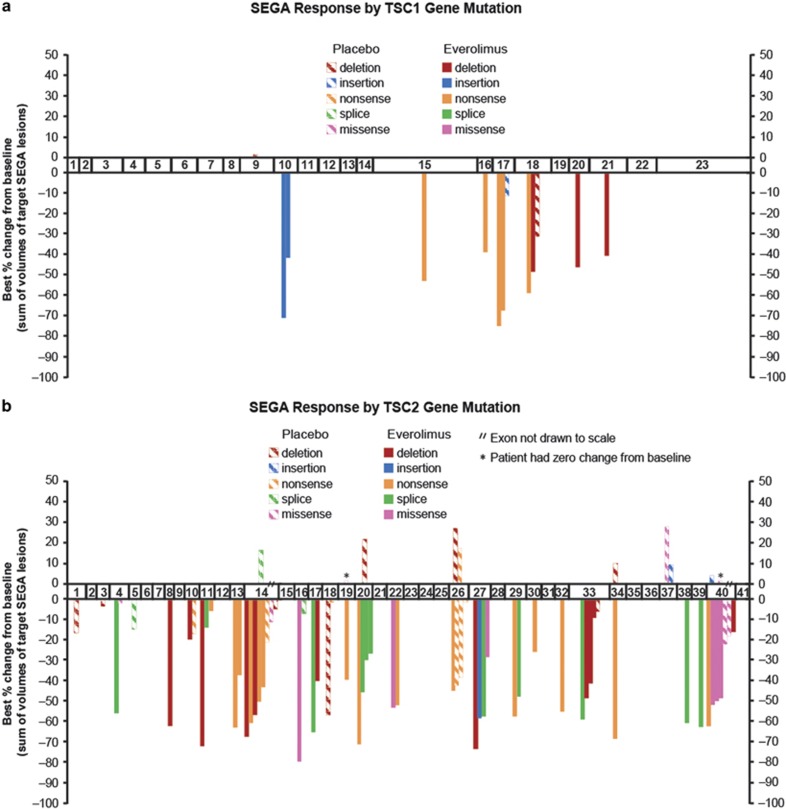

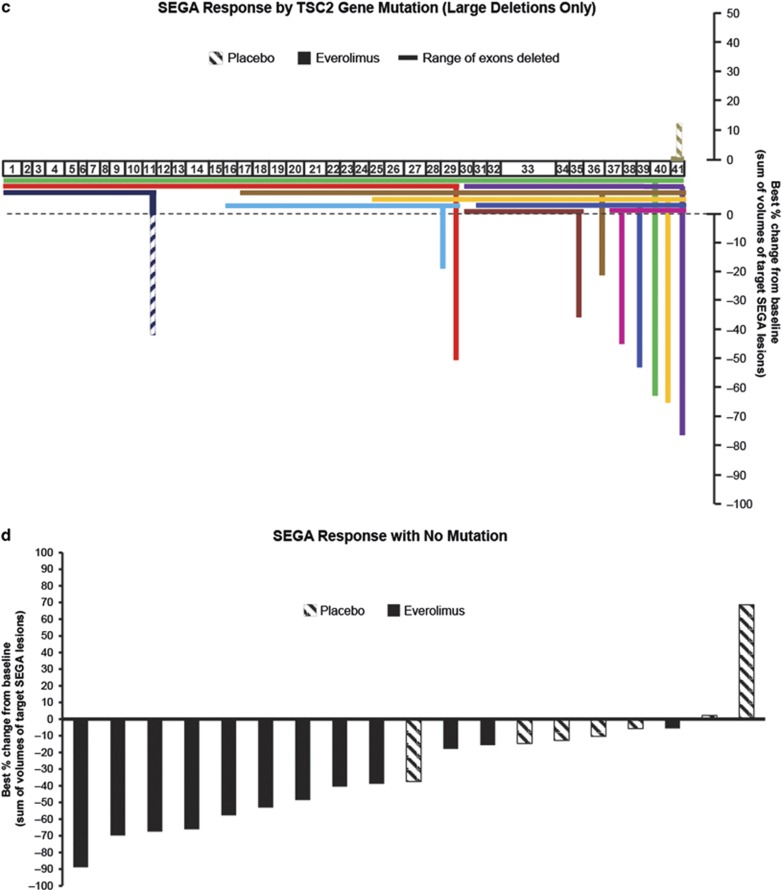
Clinical response to everolimus treatment of SEGAs in EXIST-1 according to mutation type and location in *TSC1* and *TSC2*. The best percentage change in the sum of volumes of target SEGA lesions is shown on the y axis. The mutation location and type is shown on the x axis, which is a diagram of the exons (drawn proportional to size) of *TSC1* (**a**) or *TSC2* (**b**, **c**). Note that large deletions in *TSC2* are indicated by their extent across the exons (**c**). Patients without an identified mutation are sorted by response (**d**). Subjects receiving placebo are shown with striped patterns, whereas those receiving everolimus are shown with solid patterns.

**Figure 2:**
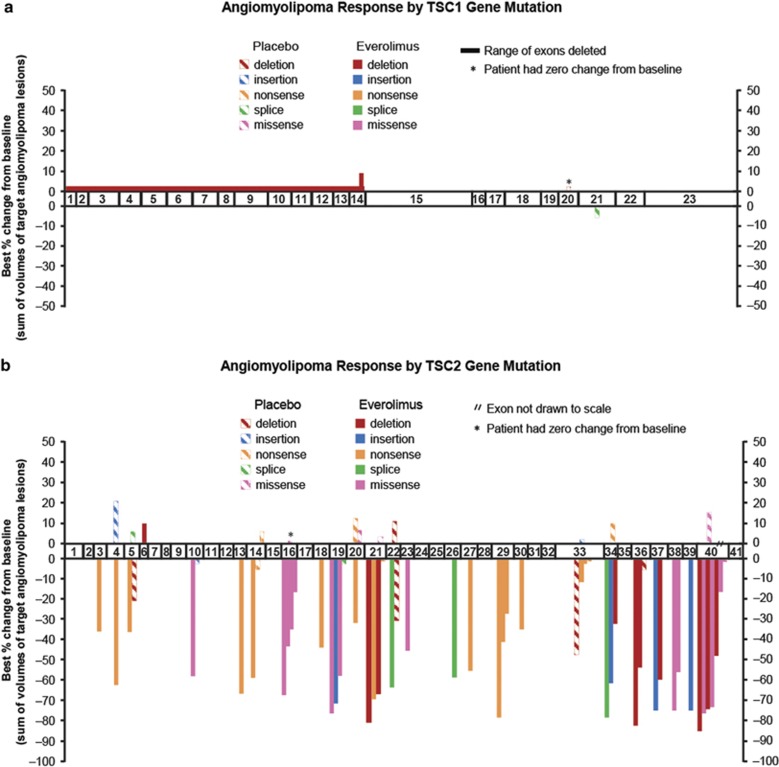

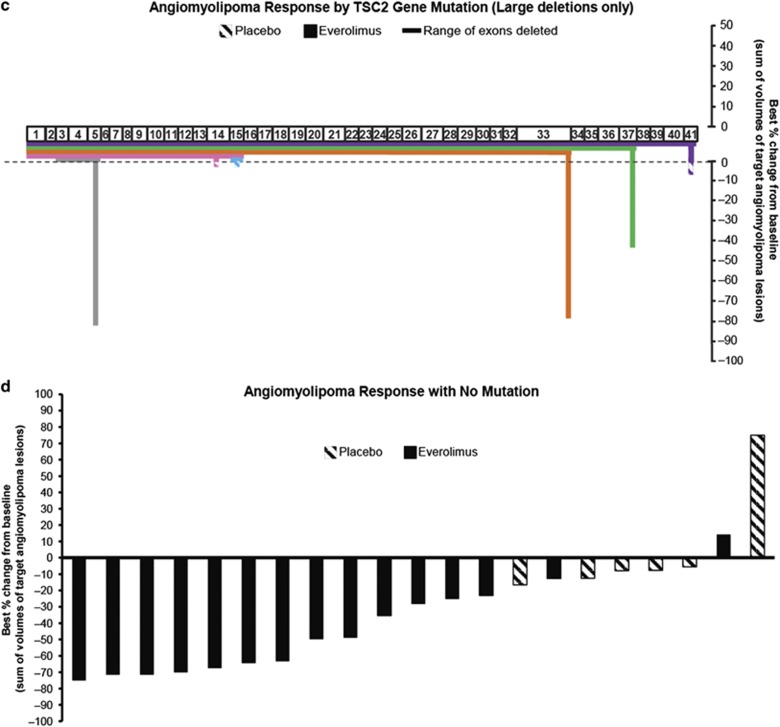
Clinical response to everolimus treatment of angiomyolipomas in EXIST-2 according to mutation type and location in *TSC1* and *TSC2*. The best percentage change in the sum of volumes of target angiomyolipoma lesions is shown on the y axis. The mutation location and type is shown on the x axis, which is a diagram of the exons (drawn proportional to size) of *TSC1* (**a**) or *TSC2* (**b** and **c**). Note that large deletions in *TSC1* and *TSC2* are indicated by their extent across the exons (**a** and **c**). Patients without an identified mutation are sorted by response (**d**). Subjects receiving placebo are shown with striped patterns, whereas those receiving everolimus are shown with solid patterns.

The site and type of mutation within *TSC1* and *TSC2* compared with response to treatment for both the everolimus- and placebo-treated patients (Figures 1 and 2) were then examined. In both EXIST-1 and EXIST-2, the location of the mutation had no apparent correlation with response. In addition, there was no significant difference in response comparing those subjects with truncating *vs* nontruncating mutations in either *TSC1* or *TSC2*, those subjects with any *TSC2* mutation *vs* those with no mutation identified or those subjects with any *TSC1* mutation *vs* those with any *TSC2* mutation for either EXIST-1 or EXIST-2 analyzed separately (all *P*>0.2, *t*-test).

## Discussion

Multiple previous genotype–phenotype studies have documented that TSC patients with *TSC2* mutations have on average more severe disease than those with *TSC1* mutations.^22, 26, 31, 37^ A meta-analysis that considered the findings from the three largest series found that subependymal nodules (a precursor lesion to SEGA), intellectual disability, seizures, facial angiofibroma, fibrous forehead plaque, renal angiomyolipoma, renal cysts and retinal phakomata or hamartomas were all significantly more common in individuals with *TSC2* mutations than in those with *TSC1* mutations.^31^ Diagnosis of renal angiomyolipoma had the highest odds ratio, 8.27 (confidence interval, 4.36–15.7), in TSC patients with *TSC2* mutations *vs* those with *TSC1* mutations, of all TSC clinical features considered.^31^ Thus, our findings here that TSC subjects with SEGA enrolled on EXIST-1 had a marginally significant increase in the proportion of *TSC2*:*TSC1* mutations, 87 *vs* 13% (*P*=0.0662), fit these previous observations. Furthermore, the extreme discordance toward *TSC2* mutation in the angiomyolipoma TSC patients enrolled in the EXIST-2 trial, 97% *TSC2* *vs* 3% *TSC1* (*P*<0.0001), also fits the high odds ratio seen for angiomyolipomas in favor of *TSC2* mutations in previous studies.^33^ Clinically, this finding translates to the simple clinical inference that TSC subjects with significant renal angiomyolipoma are much more likely to have a *TSC2* mutation than a *TSC1* mutation.

Multiple clinical studies have now confirmed the clinical benefit of everolimus and rapamycin for TSC tumors occurring in the brain, kidneys and lungs.^6, 7, 34, 35, 38, 39, 40, 41^ This includes three randomized clinical trials, including EXIST-1 and EXIST-2, all of which were positive.^34, 35, 40^ Although several studies have examined the potential for correlation between mutation site and type within *TSC1* and *TSC2* and various TSC clinical manifestations, few correlations have been seen to date; this is consistent with the model that the majority of mutations in these genes are inactivating, effectively functioning as null alleles. There are two well-documented exceptions in which there is a clear correlation between mutation and clinical phenotype. First, large genomic deletions that affect both *TSC2* and the adjacent *PKD1* gene lead to early-onset, severe polycystic kidney disease.^13^ Second, there are a number of missense mutations in *TSC2* that are associated with a relatively mild phenotype.^42, 43, 44^ However, these two types of mutation currently account for <5% of reported TSC gene mutations. Seven patients in the EXIST-1 SEGA trial had large genomic deletions extending into the *PKD1* gene (Figure 1c). None of the patients in the EXIST-2 angiomyolipoma trial had large genomic deletions extending into the *PKD1* gene (Figure 2c). None of the patients on either trial had a missense variant in *TSC2* associated with a relatively mild phenotype.

Our observations here that the site and type of mutation in either *TSC1* (in the limited number of cases available from each study; Figures 1a and 2a) or *TSC2* (Figures 1b, c, and 2b and c) appeared to have no impact on response to everolimus fits with the apparently identical pathophysiological effects of mutations throughout these genes. Nonetheless, the possibility of a correlation was important to examine carefully here because such a correlation might be important for clinical decision making and would also have broader implications for our understanding of the pathogenesis of TSC. The response rate seen in subjects without an identified mutation was similar to that of patients with defined mutations. This observation suggests that subjects without defined mutations have a similar pathogenic mechanism with mammalian target of rapamycin complex 1 activation driving tumor development. This is consistent with the hypothesis that those individuals may have unusual and difficult-to-identify mutations in *TSC1* or *TSC2*, or are mosaic for a *TSC1* or *TSC2* mutation that was missed by conventional sequencing analysis.^45, 46^

## Figures and Tables

**Table 1:** Mutation findings in EXIST-1: the SEGA trial

*Mutation type*	TSC1	TSC2	*Total*	*Percentage of 116, total number*
Deletion	5	18	23	20
In-frame deletion/insertion	0	3	3	3
Insertion	3	2	5	4
Large deletion	0	11	11	9
Missense	0	13	13	11
Nonsense	5	22	27	23
Splice	0	15	15	13
Any	13	84	97	84
No mutation identified			19	16
Total			116	
Percentage of 97 subjects with mutation identified	13	87		

**Table 2:** Mutation findings in EXIST-2: the angiomyolipoma trial

*Mutation type*	TSC1	TSC2	*Total*	*Percentage of 109, total number*
Deletion	1	11	12	11
In-frame deletion/insertion	0	5	5	5
Insertion	0	9	9	8
Large deletion	1	6	7	6
Missense	0	24	24	22
Nonsense	0	25	25	23
Splice	1	6	7	6
Any	3	86	89	82
No mutation identified			20	18
Total			109	
Percentage of 89 subjects with mutation identified	3	97		
